# Supplementary material for: Cavity Born–Oppenheimer Coupled Cluster Theory: Toward Electron Correlation in the Vibrational Strong Light-Matter Coupling Regime
Source: J Chem Theory Comput. 2025 Nov 19;21(23):12081–93. doi: 10.1021/acs.jctc.5c01604 (PMC12874383; doi:10.1021/acs.jctc.5c01604)
Supplement: Supplementary file 1 [file ct5c01604_si_001.pdf]

# Supporting Information – Cavity Born-Oppenheimer Coupled Cluster Theory: Towards Electron Correlation in the Vibrational Strong Light-Matter Coupling Regime

TABLE I. Menshutkin reactant coordinates (Å), cavity-induced reorientation RHF

| | | | |
|---|---|---|---|
| C | -0.067775 | 1.131563 | -3.852424 |
| H | -0.597912 | 0.242070 | -3.526028 |
| H | 0.959961 | 1.121745 | -3.503769 |
| H | -0.581526 | 2.029703 | -3.523575 |
| Br | -0.044632 | 1.133979 | -5.811100 |
| N | -0.002106 | 1.140608 | -0.714204 |
| C | 0.000000 | 0.000000 | 0.000000 |
| C | 0.003739 | -0.038914 | 1.394714 |
| C | 0.005447 | 1.165307 | 2.097804 |
| C | 0.003502 | 2.357001 | 1.373679 |
| C | -0.000223 | 2.293576 | -0.020131 |
| H | 0.008407 | 1.174852 | 3.184806 |
| H | -0.000867 | -0.921723 | -0.579349 |
| H | 0.005346 | -0.993282 | 1.913710 |
| H | 0.004923 | 3.320336 | 1.875833 |
| H | -0.001232 | 3.205006 | -0.61556 |

TABLE II. Menshutkin transition state coordinates (Å), cavity-induced reorientation RHF

| | | | |
|---|---|---|---|
| C | -0.014650 | 1.166080 | -2.510997 |
| H | -1.086196 | 1.165880 | -2.621464 |
| H | 0.520378 | 2.092127 | -2.641678 |
| H | 0.520725 | 0.240236 | -2.641688 |
| Br | -0.041498 | 1.166070 | -5.030292 |
| N | 0.012198 | 1.166077 | -0.666676 |
| C | 0.000000 | 0.000000 | 0.000000 |
| C | -0.024620 | -0.036255 | 1.391040 |
| C | -0.036686 | 1.166011 | 2.099090 |
| C | -0.024624 | 2.368311 | 1.391116 |
| C | -0.000005 | 2.332122 | 0.000073 |
| H | -0.055171 | 1.165985 | 3.185256 |
| H | 0.009723 | -0.900480 | -0.609358 |
| H | -0.032655 | -0.992423 | 1.905088 |
| H | -0.032662 | 3.324455 | 1.905224 |
| H | 0.009719 | 3.232630 | -0.609229 |

TABLE III. Menshutkin product coordinates (Å), cavity-induced reorientation RHF

| | | | |
|---|---|---|---|
| C | -0.003047 | 1.175232 | -2.173818 |
| H | 0.505032 | 0.287898 | -2.538344 |
| H | -1.032865 | 1.177594 | -2.524563 |
| H | 0.509318 | 2.059682 | -2.539360 |
| Br | -0.104707 | 1.176733 | -5.140193 |
| N | 0.013482 | 1.175950 | -0.663284 |
| C | 0.000000 | 0.000000 | 0.000000 |
| C | -0.032194 | -0.023892 | 1.387670 |
| C | -0.047220 | 1.177696 | 2.095493 |
| C | -0.032140 | 2.378400 | 1.386144 |
| C | 0.000067 | 2.352754 | -0.001480 |
| H | -0.070243 | 1.178383 | 3.181160 |
| H | 0.012609 | -0.892360 | -0.616773 |
| H | -0.040770 | -0.981052 | 1.898370 |
| H | -0.040674 | 3.336205 | 1.895633 |
| H | 0.012724 | 3.244312 | -0.619429 |

TABLE IV. Menshutkin reactant coordinates (Å), cavity-induced reorientation CCSD

| | | | |
|---|---|---|---|
| C | 0.044622 | -1.132198 | -3.852548 |
| H | 0.568566 | -0.237219 | -3.531185 |
| H | -0.979979 | -1.132747 | -3.494652 |
| H | 0.570460 | -2.024931 | -3.528098 |
| Br | 0.003862 | -1.135553 | -5.810923 |
| N | 0.007321 | -1.140787 | -0.713879 |
| C | 0.000000 | 0.000000 | 0.000000 |
| C | 0.008427 | 0.039374 | 1.394672 |
| C | 0.025352 | -1.164553 | 2.098072 |
| C | 0.032944 | -2.356427 | 1.374287 |
| C | 0.023465 | -2.293468 | -0.019508 |
| H | 0.032281 | -1.173737 | 3.185053 |
| H | -0.013766 | 0.921479 | -0.579580 |
| H | 0.001749 | 0.993865 | 1.913399 |
| H | 0.045885 | -3.319549 | 1.876693 |
| H | 0.028419 | -3.205056 | -0.614675 |

TABLE V. Menshutkin transition state coordinates (Å), cavity-induced reorientation CCSD

| | | | |
|---|---|---|---|
| C | 0.066816 | 1.048982 | -2.561323 |
| H | -1.001247 | 1.054030 | -2.701457 |
| H | 0.615307 | 1.962794 | -2.719422 |
| H | 0.595419 | 0.112963 | -2.634428 |
| Br | 0.108923 | 0.932907 | -5.077753 |
| N | 0.043179 | 1.133877 | -0.718898 |
| C | 0.000000 | 0.000000 | 0.000000 |
| C | -0.063073 | 0.028252 | 1.390024 |
| C | -0.081375 | 1.261988 | 2.041544 |
| C | -0.036805 | 2.430142 | 1.279740 |
| C | 0.025476 | 2.329526 | -0.106963 |
| H | -0.129577 | 1.312292 | 3.125639 |
| H | 0.016555 | -0.927706 | -0.566885 |
| H | -0.095621 | -0.903037 | 1.946996 |
| H | -0.048462 | 3.409036 | 1.749004 |
| H | 0.061710 | 3.200804 | -0.756450 |

TABLE VI. Menshutkin product coordinates (Å), cavity-induced reorientation CCSD

| | | | |
|---|---|---|---|
| C | -0.001756 | 1.192474 | -2.164408 |
| H | -0.472897 | 0.291247 | -2.544154 |
| H | 1.032988 | 1.233071 | -2.497878 |
| H | -0.538224 | 2.061789 | -2.531676 |
| Br | 0.148494 | 1.221849 | -5.128583 |
| N | -0.043105 | 1.180207 | -0.654396 |
| C | 0.000000 | 0.000000 | 0.000000 |
| C | 0.010204 | -0.034179 | 1.387795 |
| C | -0.027816 | 1.161358 | 2.104952 |
| C | -0.072615 | 2.366628 | 1.404661 |
| C | -0.081125 | 2.351299 | 0.016533 |
| H | -0.022663 | 1.153910 | 3.190829 |
| H | 0.028283 | -0.887167 | -0.623714 |
| H | 0.043373 | -0.994646 | 1.891225 |
| H | -0.105463 | 3.319941 | 1.921542 |
| H | -0.114350 | 3.246947 | -0.594699 |

TABLE VII. MeOH@1$H_2O$ cluster (Å), cavity-induced reorientation RHF

| | | | |
|---|---|---|---|
| O | 0.000000 | 0.000000 | 0.000000 |
| H | 0.377018 | 0.426125 | 0.780560 |
| C | 0.579243 | 0.622018 | -1.128930 |
| H | 0.181998 | 0.130008 | -2.012759 |
| H | 1.667189 | 0.523660 | -1.147773 |
| H | 0.331424 | 1.684247 | -1.193989 |
| O | 1.182978 | 1.229169 | 2.321051 |
| H | 0.528343 | 1.609934 | 2.912108 |
| H | 1.608798 | 0.548591 | 2.848778 |

TABLE VIII. MeOH fragment of MeOH@1$H_2O$ cluster (Å), cavity-induced reorientation RHF

| | | | |
|---|---|---|---|
| O | 0.000000 | 0.000000 | 0.000000 |
| H | 0.377018 | 0.426125 | 0.780560 |
| C | 0.579243 | 0.622018 | -1.128930 |
| H | 0.181998 | 0.130008 | -2.012759 |
| H | 1.667189 | 0.523660 | -1.147773 |
| H | 0.331424 | 1.684247 | -1.193989 |

TABLE IX. 1$H_2O$ fragment of MeOH@1$H_2O$ cluster (Å), cavity-induced reorientation RHF

| | | | |
|---|---|---|---|
| O | 1.182978 | 1.229169 | 2.321051 |
| H | 0.528343 | 1.609934 | 2.912108 |
| H | 1.608798 | 0.548591 | 2.848778 |

TABLE X. MeOH@1$H_2O$ cluster (Å), cavity-induced reorientation CCSD

| | | | |
|---|---|---|---|
| O | 0.000000 | 0.000000 | 0.000000 |
| H | -0.083146 | 0.558988 | 0.783332 |
| C | -0.102004 | 0.849402 | -1.124731 |
| H | 0.018523 | 0.233007 | -2.011608 |
| H | 0.674308 | 1.618022 | -1.139480 |
| H | -1.073948 | 1.344740 | -1.187637 |
| O | -0.178344 | 1.685061 | 2.329431 |
| H | -0.891559 | 1.426902 | 2.918969 |
| H | 0.616317 | 1.569459 | 2.856848 |

TABLE XI. MeOH fragment of MeOH@1$H_2O$ cluster (Å), cavity-induced reorientation CCSD

| | | | |
|---|---|---|---|
| O | 0.000000 | 0.000000 | 0.000000 |
| H | -0.083146 | 0.558988 | 0.783332 |
| C | -0.102004 | 0.849402 | -1.124731 |
| H | 0.018523 | 0.233007 | -2.011608 |
| H | 0.674308 | 1.618022 | -1.139480 |
| H | -1.073948 | 1.344740 | -1.187637 |

TABLE XII. 1$H_2O$ fragment of MeOH@1$H_2O$ cluster (Å), cavity-induced reorientation CCSD

| | | | |
|---|---|---|---|
| O | -0.178344 | 1.685061 | 2.329431 |
| H | -0.891559 | 1.426902 | 2.918969 |
| H | 0.616317 | 1.569459 | 2.856848 |

TABLE XIII. MeOH@5$H_2$O cluster (Å), cavity-induced reorientation RHF

| | | | |
|---|---|---|---|
| O | 0.000000 | 0.000000 | 0.000000 |
| H | 0.698090 | -0.545899 | -0.384282 |
| C | 0.590116 | 1.234364 | 0.353563 |
| H | -0.194158 | 1.866434 | 0.761536 |
| H | 1.026898 | 1.749696 | -0.505103 |
| H | 1.365207 | 1.123967 | 1.115810 |
| O | 2.493247 | -1.913077 | -1.460515 |
| H | 2.618266 | -2.755853 | -1.016705 |
| H | 2.220259 | -2.153670 | -2.349670 |
| H | -0.343301 | -0.647094 | 2.390264 |
| O | -0.333405 | -1.020406 | 3.263779 |
| H | -0.984795 | -0.585501 | 3.801418 |
| H | -1.761155 | 0.695283 | -1.924798 |
| O | -2.402684 | 0.960993 | -2.573128 |
| H | -2.162744 | 0.604828 | -3.420521 |
| O | 2.535730 | 2.456598 | 2.240058 |
| H | 3.375972 | 2.056531 | 2.430954 |
| H | 2.505092 | 3.326167 | 2.621382 |
| H | 1.839807 | 2.668579 | -2.342957 |
| O | 2.107069 | 3.227619 | -2.182830 |
| H | 2.466829 | 3.640500 | -2.959100 |

TABLE XIV. MeOH fragment of MeOH@5$H_2$O cluster (Å), cavity-induced reorientation RHF

| | | | |
|---|---|---|---|
| O | 0.000000 | 0.000000 | 0.000000 |
| H | 0.698090 | -0.545899 | -0.384282 |
| C | 0.590116 | 1.234364 | 0.353563 |
| H | -0.194158 | 1.866434 | 0.761536 |
| H | 1.026898 | 1.749696 | -0.505103 |
| H | 1.365207 | 1.123967 | 1.115810 |

TABLE XV. 5$H_2$O fragment of MeOH@5$H_2$O cluster (Å), cavity-induced reorientation RHF

| | | | |
|---|---|---|---|
| O | 2.493247 | -1.913077 | -1.460515 |
| H | 2.618266 | -2.755853 | -1.016705 |
| H | 2.220259 | -2.153670 | -2.349670 |
| H | -0.343301 | -0.647094 | 2.390264 |
| O | -0.333405 | -1.020406 | 3.263779 |
| H | -0.984795 | -0.585501 | 3.801418 |
| H | -1.761155 | 0.695283 | -1.924798 |
| O | -2.402684 | 0.960993 | -2.573128 |
| H | -2.162744 | 0.604828 | -3.420521 |
| O | 2.535730 | 2.456598 | 2.240058 |
| H | 3.375972 | 2.056531 | 2.430954 |
| H | 2.505092 | 3.326167 | 2.621382 |
| H | 1.839807 | 2.668579 | -2.342957 |
| O | 2.107069 | 3.227619 | -2.182830 |
| H | 2.466829 | 3.640500 | -2.959100 |

TABLE XVI. MeOH@5$H_2O$ cluster (Å), cavity-induced reorientation CCSD

| | | | |
|---|---|---|---|
| O | 0.000000 | 0.000000 | 0.000000 |
| H | 0.782057 | -0.398529 | -0.403204 |
| C | 0.317499 | 1.350826 | 0.267140 |
| H | -0.566468 | 1.815591 | 0.695674 |
| H | 0.585396 | 1.903514 | -0.636421 |
| H | 1.134656 | 1.453996 | 0.985181 |
| O | 2.780611 | -1.392338 | -1.529347 |
| H | 3.111943 | -2.162346 | -1.060120 |
| H | 2.524120 | -1.733686 | -2.389986 |
| H | -0.071373 | -0.579595 | 2.430829 |
| O | 0.064780 | -0.894929 | 3.316558 |
| H | -0.639481 | -0.586150 | 3.874396 |
| H | -1.965223 | 0.188319 | -1.841835 |
| O | -2.681229 | 0.271826 | -2.460587 |
| H | -2.410509 | -0.067095 | -3.305777 |
| O | 2.033745 | 3.068882 | 1.982052 |
| H | 2.950372 | 2.874042 | 2.138049 |
| H | 1.829392 | 3.929319 | 2.329019 |
| H | 1.081010 | 2.880301 | -2.555571 |
| O | 1.224805 | 3.492087 | -2.434584 |
| H | 1.444603 | 3.932391 | -3.247182 |

TABLE XVII. MeOH fragment of MeOH@5$H_2O$ cluster (Å), cavity-induced reorientation CCSD

| | | | |
|---|---|---|---|
| O | 0.000000 | 0.000000 | 0.000000 |
| H | 0.782057 | -0.398529 | -0.403204 |
| C | 0.317499 | 1.350826 | 0.267140 |
| H | -0.566468 | 1.815591 | 0.695674 |
| H | 0.585396 | 1.903514 | -0.636421 |
| H | 1.134656 | 1.453996 | 0.985181 |

TABLE XVIII. 5$H_2O$ fragment of MeOH@5$H_2O$ cluster (Å), cavity-induced reorientation CCSD

| | | | |
|---|---|---|---|
| O | 2.780611 | -1.392338 | -1.529347 |
| H | 3.111943 | -2.162346 | -1.060120 |
| H | 2.524120 | -1.733686 | -2.389986 |
| H | -0.071373 | -0.579595 | 2.430829 |
| O | 0.064780 | -0.894929 | 3.316558 |
| H | -0.639481 | -0.586150 | 3.874396 |
| H | -1.965223 | 0.188319 | -1.841835 |
| O | -2.681229 | 0.271826 | -2.460587 |
| H | -2.410509 | -0.067095 | -3.305777 |
| O | 2.033745 | 3.068882 | 1.982052 |
| H | 2.950372 | 2.874042 | 2.138049 |
| H | 1.829392 | 3.929319 | 2.329019 |
| H | 1.081010 | 2.880301 | -2.555571 |
| O | 1.224805 | 3.492087 | -2.434584 |
| H | 1.444603 | 3.932391 | -3.247182 |
